# Examining the psychometric properties of a sport-related concussion survey: a Rasch measurement approach

**DOI:** 10.1186/s13104-017-2559-z

**Published:** 2017-06-26

**Authors:** Mark Hecimovich, Ida Marais

**Affiliations:** 1 Division of Athletic Training, University of Northern Iowa, 003C Human Performance Center, Cedar Falls, IA USA; 2 School of Psychology and Exercise Science, Murdoch University, South Street, Murdoch, WA Australia; 3 University of Western Australia, Graduate School of Education, M428, 35 Stirling Highway, Crawley, WA Australia

**Keywords:** Concussion awareness, Measurement, Rasch model, Education

## Abstract

**Background:**

Awareness of sport-related concussion (SRC) is an essential step in increasing the number of athletes or parents who report on SRC. This awareness is important, as there is no established data on medical care at youth-level sports and may be limited to individuals with only first aid training. In this circumstance, aside from the coach, it is the players and their parents who need to be aware of possible signs and symptoms. The aim of this study was to examine the psychometric properties of a parent and player concussion survey intended for use before and after an education campaign regarding SRC.

**Methods:**

1441 questionnaires were received from parents and 284 questionnaires from players. The responses to the sixteen-item section of the questionnaire’s ‘recognition of signs and symptoms’ were submitted to psychometric analysis using the dichotomous and polytomous Rasch model via the Rasch Unidimensional Measurement Model software RUMM2030. The Rasch model of Modern Test Theory can be considered a refinement of, or advance on, traditional analyses of an instrument’s psychometric properties.

**Results:**

The main finding is that these sixteen items measure two factors: items that *are* symptoms of concussion and items that *are not* symptoms of concussion. Parents and athletes were able to identify most or all of the symptoms, but were not as good at distinguishing symptoms that *are not* symptoms of concussion. Analyzing these responses revealed differential item functioning for parents and athletes on non-symptom items. When the DIF was resolved a significant difference was found between parents and athletes.

**Conclusions:**

The main finding is that the items measure two ‘dimensions’ in concussion symptom recognition. The first dimension consists of those items that* are* symptoms of concussion and the second dimension of those items that *are not* symptoms of concussion. Parents and players were able to identify most or all of the symptoms of concussion, so one would not expect to pick up any positive change on these items after an education campaign. Parents and players were not as good at distinguishing symptoms that *are not* symptoms of concussion. It is on these items that one may possibly expect improvement to manifest, so to evaluate the effectiveness of an education campaign it would pay to look for improvement in distinguishing symptoms that *are not* symptoms of concussion.

## Background

Sport-related concussion (SRC) is referred to as an ‘epidemic’ by the Centers for Disease Control and Prevention [1]. Collision sports such as American football, rugby and Australian football (AF) are purported to have high rates of SRC at the adult, junior and youth level [2–5]. This is a concern for young athletes as their developing brains have been shown to be more vulnerable and to have poorer outcomes following SRC than adults [6, 7].

The prevention of SRC in children and adolescents is important from a public health perspective, given the number of individuals participating in organized youth sports [8, 9]. Alongside primary prevention (i.e., reducing the incidence of concussions), secondary prevention (i.e., early identification of potential concussions and subsequent removal from play) is a critical element of risk reduction. During the symptomatic period that occurs post-concussion, there appears to be a temporal window of vulnerability when an additional impact often means magnified neurologic consequences [10, 11].

The diagnosis of SRC is not straightforward, given the absence of a specific diagnostic test or biomarker for SRC. Therefore, the current cornerstone of SRC diagnosis is confirming the presence of a constellation of signs and symptoms after an individual has experienced a hit to the head or body [12] through self-report SRC scales. SRC symptom scales assess a broad range of symptoms considered pathognomonic of concussion. Of the approximately 20 different symptom scales commonly used to evaluate concussion, 14 are variations of 6 core scales [13], which include symptoms associated with SRC [13, 14] and other components such as recognition of signs and symptoms, SRC management, and return-to-play.

Unfortunately, few self-report concussion scales are developed systematically or had published psychometric properties [13, 14]. Gioia et al.’s [15] literature review of concussion symptom scales used in the paediatric, adolescent, and young adult populations (ages 5–22 years) identified one research-based scale and four scales that were in clinical use; psychometric evidence for the scales was assessed through traditional methods for younger children (5–12 years) and for adolescents and young adults (13–22 years).

Sport-related concussion assessment during competition or practice is facilitated by the presence of a certified athletic trainer, team physician, or other health care provider at the venue (e.g., field, gymnasium, or rink) where the injury occurred. However, the vast majority of young athletes practice and play in circumstances where trained personnel are not routinely available to make sideline injury assessments, and the responsibility for determining whether to remove an athlete from play falls on coaches, parents, players, and, perhaps, officials [12]. Furthermore, symptoms may not become apparent for several hours after injury, and one result of this is that a large number of SRC are not identified until 24 h or more after the injury [16, 17]. Therefore, educational interventions in order to build player and parent awareness of SRC signs and symptoms are imbedded in many youth and junior-level sporting organisations [18–23]. The use of SRC symptom scales are used in these interventions and it is therefore vital they are valid and reliable. This study utilized the Rasch measurement theory [24] to examine the psychometric properties of a questionnaire intended to measure AF players’ and their parents’ awareness of SRC [25]. Because the questionnaire is to be used to assess change in players’ and their parents’ awareness of concussion symptoms after a targeted education campaign it needs to be valid, reliable and able to detect clinically and statistically significant change.

The Rasch model of Modern Test Theory (1960/1980) [24] can be considered a refinement of, or advance on, traditional analyses of an instrument’s psychometric properties [26]. It is a model for analyzing observations that are manifestations of the trait to be measured. The trait is latent, which means it is not directly observable, for example awareness of concussion. It is used where responses to a number of items are intended to be summed to provide a summative ‘measure’ for each person. Hobart and Cano [26] have demonstrated a number of refinements that result from using the Rasch model to establish the psychometric properties of scales. They described the results of five studies, which assessed different aspects of measurement and used different scales, and concluded that there is considerable benefit in using Rasch analysis in addition to traditional psychometric methods in health measurement.

Findings from each of the five studies show that Rasch analysis is empirically superior to traditional psychometric methods for evaluating rating scales, developing rating scales, analysing rating scale data, understanding and measuring stability and change, and understanding the health constructs we seek to quantify (Hobart and Cano 2009, p iii) [26].

In Rasch analysis estimates for items and persons are on the same scale, which allows for an examination of item/person alignment, typically referred to as item/person ‘targeting’. Rasch analysis evaluates ‘differential item functioning’ that ensures uniformity and stability of the scale across different population groups at all difficulty levels. In addition, Rasch analysis examines response categories to ensure that each response option is ordered and should reflect increasing levels of the latent trait being measured [27].

Whilst the Rasch paradigm is being increasingly used in the development and evaluation of clinical tools in health and medical sciences, including rehabilitation and exercise science, psychology, nursing and podiatry [28–32], it is relatively novel in concussion awareness research [14].

## Methods

### Instrument

The questionnaire, developed by Gourley et al. [25] in their study of concussion awareness in youth and parents affiliated with American football, was slightly modified from a previously published survey [33]. Slight amendments were made for use in Australia, such as use of Australian terminology. Prior to administration, a pilot study was performed using 7 youth AF players (M = 14.5 years old) and 6 parents of current AF youth football players to assess clarity and provide feedback; no additional changes were suggested. The revised questionnaire consists of demographic questions, and questions on three areas which have been reported to be important aspects of SRC awareness, (1) recognition of signs and symptoms, (2) concussion management, and (3) return-to-play [33–35]. Because only items from “Background” section are intended to be summed to provide a total score for each person, these items were analyzed with the Rasch model and this paper describes the results. Sixteen items formed the recognition of signs and symptoms (S&S) section and include the most commonly reported signs and symptoms of SRC as well as some symptoms that are *not* symptoms of concussion [36, 37]. Response categories were *Yes*, *No*, and *Don’t Know*.

The demographic questions for parents and players were different. The parent demographic section included items on how many years their children have played organised, coached, sports, what percentage of the games/competitions they attend, and level of education. Also included were items aimed at determining their level of concussion awareness such as whether they had first aid training, and training specific for concussion. The player demographic section included items on years participating in football, location (metropolitan, regional), and if they had ever had their ‘bell rung’ when playing football (a slang nomenclature term used when sustaining a hit to the head) [38]. No item pertained to previous concussion training was included as this was not the current policy at that time to have this available to players.

### Participants

The institutions Human Research Ethics Committee approved this study (2015/2017) and there was full support of the West Australia Football Commission (WAFC). An on-line survey that comprised the scale with different demographic questions for parents and players was linked into the West Australia Football Commission (WAFC) web site and made available to all registered active youth football players and parents of current registered youth football players. The exclusion criteria were incomplete surveys. The surveys were available from March through July 2015. This period was chosen as it occurred during the participation season when players and parents are actively involved in the sport and likely to access the web site. All players and parents were notified by the WAFC about the surveys via e-mail prior to and at the launch of the web site link. Periodic notices were sent to all registered parents and youth football players during the web site link availability. Approximately 6600 parents and 4100 players had access to the survey, however not all registered active parents and players continued to participate over the 2015 season with dropouts occurring periodically.

### Analysis

A total of *n* = 1441 questionnaires were received from parents and *n* = 284 questionnaires from players resulting in a total sample of *n* = 1725 questionnaires. Responses to the questionnaires were submitted to psychometric analysis using the dichotomous and polytomous Rasch model [24] via the Rasch Unidimensional Measurement Model software RUMM2030 [39]. The dichotomous model is used where there are only two response categories. When there are three or more response categories the polytomous Rasch model is used.

#### Scoring of response categories

The data were scored in three different ways and then analysed::

I. First, the responses were scored dichotomously with *Incorrect* = 0 and *Correct* = 1 and *Don’t know* scored as *Incorrect* (0). The data were then analysed using the dichotomous Rasch model.
II. Second, the items were scored polytomously, that is, *Incorrect* = 0, *Don’t know* = 1, and *Correct* = 2. That way, respondents get some credit for being aware they don’t know whether something is a symptom, rather than identifying it as a symptom incorrectly. With this scoring method the data were analysed with the polytomous Rasch model.
III. Third, *Don’t know* was scored as missing data and responses analysed using the dichotomous model. The Rasch model routinely handles missing data.

An analysis of responses according to the Rasch model refines results from traditional analyses in a number of ways. The analysis focused on these aspects:

(i) *Item difficulty and person estimates* Because Rasch item (difficulty to endorse) and person estimates are on the same scale the alignment of items to persons can be assessed.
(ii) *Item invariance across subgroups of the population* Comparisons between the summed scores on an instrument for members of subgroups can be misleading if, for the same level of the trait being measured, members of the different subgroups respond differently to individual items [40]. Rasch analysis has sophisticated graphical and statistical ways differential item functioning (DIF) can be identified.
(iii) *Unidimensionality* If responses to different items are to be summed it is important that they measure the same thing. In Factor Analysis items that are very difficult to endorse compared to other items can sometimes lead to other ‘factors’ that emerge from the analysis [41]. Because ‘difficulty to endorse’ is taken into account in the Rasch model, the ‘factors’ or ‘dimensions’ that emerge do not reflect difficulty but can be taken to be substantive dimensions of the instrument.
(iv) *Response categories* In traditional analyses it is assumed that response categories functioned as intended. In Rasch analysis response category functioning is examined to ensure that each successive response option reflect increasing levels of the latent trait being measured.

## Results

### Frequencies of responses in each response category

There were no missing responses. Table 1 shows the frequencies of responses (as a percentage) in each response category. The items in bold *are* symptoms of concussion so the correct response to these items is *Yes* and the correct response to the non-bold items is *No*.

**Table 1:** Frequencies of responses (as a percentage) in each response category

Item	Statement	No	Don’t know	Yes
1	Abnormal sense of smell	0.32	0.41	0.27
2	Abnormal sense of taste	0.30	0.44	0.26
*3*	*Loss of memory*	*0.03*	*0.01*	*0.95*
*4*	*Blurred vision*	*0.02*	*0.01*	*0.97*
5	Chest pain	0.07	0.31	0.63
*6*	*Dizziness*	*0.01*	*0.01*	*0.98*
*7*	*Confusion*	*0.02*	*0.01*	*0.97*
*8*	*Headache*	*0.02*	*0.01*	*0.97*
9	Nosebleed	0.46	0.26	0.28
*10*	*Loss of consciousness*	*0.03*	*0.01*	*0.96*
11	Sharp burning pain in neck	0.38	0.39	0.23
*12*	*Nausea*	*0.04*	*0.05*	*0.91*
13	Numbness/tingling in arms/hands	0.44	0.34	0.22
14	Weakness of neck range of motion	0.49	0.31	0.19
*15*	*Sleep disturbances*	*0.12*	*0.23*	*0.65*
*16*	*Problems studying or doing class work*	*0.09*	*0.21*	*0.70*

Preliminary analyses indicated that with scoring option III (*Don’t know* scored as missing data) the reliability was considerably lower than the other two options, because of the missing responses. Cronbach’s alpha could not be calculated because of the missing values. The Rasch Person Separation Index of reliability [42] was very low at 0.25. Under conditions where there are no floor or ceiling effects the PSI is equivalent to Cronbach’s alpha [42]. The results from scoring the responses polytomously (II) were quite similar to the results with dichotomous scoring (I). Below the results for dichotomous scoring are described, and following that the results are compared with the results of polytomous scoring.

### Person/item location alignment and reliability

**Fig. 1:**
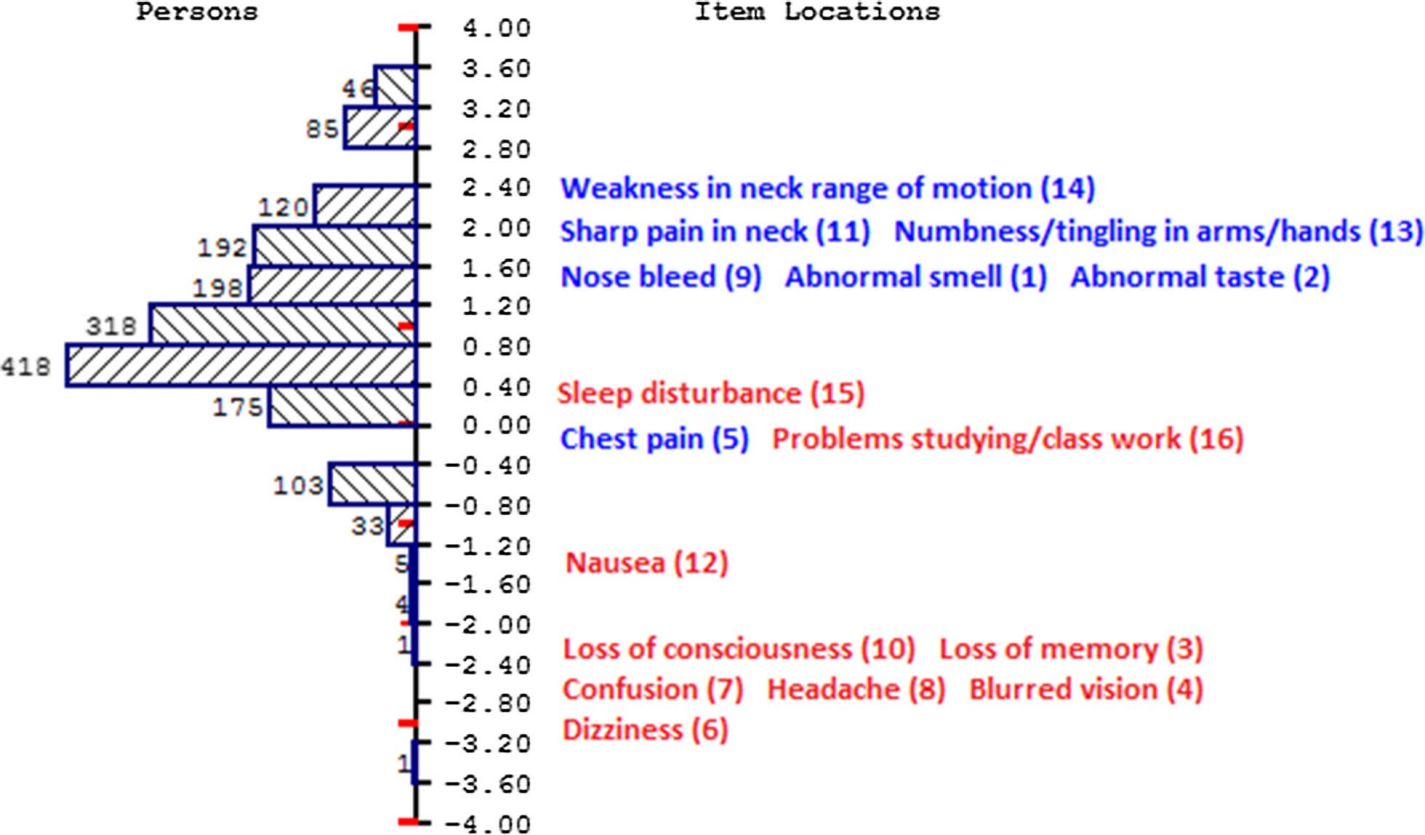
Histogram of person locations (*left*) and labeled items (*right*) on the same measurement continuum. Item labels in *blue* are the items that *are not* symptoms of concussion, and labels in *red* are of items that *are* symptoms of concussion

Figure 1 shows two frequency histograms on the same scale, one of the Rasch person locations (left) and the other of the sixteen symptom item locations (right). It is clear from the figure that the items aligned fairly well with the persons, but there were some ‘gaps’ on the measurement continuum where there is a lack of items. The mean of the person locations was 1.086 and the standard deviation (SD) was 1.077. Relative to the items, which have a constrained mean of 0, the person mean is positive, indicating that, on the whole, the questionnaire respondents were reasonably aware of concussion symptoms.

Item labels in blue are the items that *are not* symptoms of concussion, and labels in red are of items that *are* symptoms of concussion. The most difficult items to get correct were items 1 *Abnormal smell*, 2 *Abnormal taste*, 9 *Nose bleed*, 11 *Sharp pain in neck*, 13 *Numbness/tingling in arms/hands* and 14 *Weakness in neck range of motion*. It is noted that these are the items that are *not* symptoms of concussion (blue items on the graph). Respondents found these items more difficult to get correct than the items that *are* symptoms of concussion (red items on the graph), that is, items 3 *Loss of memory*, 4 *Blurred vision*, 6 *Dizziness*, 7 *Confusion*, 8 *Headache*, 10 *Loss of consciousness* and 12 *Nausea*. Items 5 *Chest pain*, 15 *Sleep disturbance* and 16 *Problems studying/class work* were of moderate difficulty.

Cronbach’s Alpha was 0.62 and The Rasch Person Separation Index of reliability was lower at 0.56. Under conditions where there are no floor or ceiling effects the PSI is equivalent to Cronbach’s alpha [42]. There are no floor or ceiling effects in these data, as evidenced by the normal person distribution in Fig. 1. Another possible reason for a discrepancy between Cronbach’s alpha and the PSI is multidimensionality. Marais and Andrich [43] showed that, when there is more than one dimension in the data PSI is reduced. In the next two sections results of tests of dimensionality as well as data fit to the model are reported.

### Fit of data to the model

Summary fit statistics indicated that the persons fitted the model reasonably well, but the items did not fit the model.

*Person fit* Person fit to the model is assessed through the person fit residual statistic, a transformed sum of individual item/person residuals for each person. The mean fit residual for the persons was −0.835 and the SD was 0.983. If data fit the model then the mean is close to 0 and the SD close to 1. Even though there were 6 persons with person fit residuals outside the range that indicate fit (|2.5|), the results did not change considerably when these persons were deleted from the analysis, so all person responses were retained.

*Item fit* Two item fit statistics were examined: the Chi square and item fitresidual fit statistics. Because the power to detect misfit increases with sample size, the sample size in the calculation of the Chi square statistic was adjusted to a realistic size for studying model fit in an instrument with 16 dichotomous items (*n* = 320). The overall item/trait Chi Square statistic (χ2 = 289.67, *df* = 64), *p* < 0.001), indicated that the data did not fit the model. The second fit statistic, the item fit residual statistic is a transformed sum of individual item/person residuals for each item and is analogous to the ‘outfit’ fit statistic typically reported by other Rasch analysis software packages. Model fit is indicated when the mean of the item fit residuals is close to 0, and the SD close to 1. The mean item fit residual was −1.650 and the SD was 3.003 indicating the items did not fit the model.

#### Dimensionality

Because these 16 items are intended to be summed to provide a total score for each person, it is important to determine their dimensionality. Firstly, a principal component analysis (PCA) of the item residuals revealed that the Eigenvalue of the first principal component (3.984) was considerably larger relative to the Eigenvalue of the second (1.740) and third principal components (1.386). Items 1, 2, 5, 9, 11, 13 and 14 (items that are *not* symptoms of concussion) had high positive loadings on the first component, indicating that these items form a second factor, whereas items 3, 4, 6, 7, 8, 10, 12, 15 and 16 had negative loadings.

Secondly, since the PCA indicated that more than one dimension or factor is present in the data a further analysis was conducted. A *t* test allows comparison of the person estimates which are derived from the two subsets of items identified in the PCA [44]. Two subsets were formed (Subset 1: items 1, 2, 5, 9, 11, 13, 14 and subset 2: 3, 4, 6, 7, 8, 10, 12, 15, 16). The results showed that 156 (9.04%) of persons had significantly different estimates on these two subsets at the 5% level of significance, and 95 (5.51%) persons had significantly different estimates at the 1% level. Since more than 5% (at 5% level of significance) and more than 1% (at 1% level of significance) of persons obtained significantly varying person estimates when compared on the two subtests, multidimensionality can be inferred. In Factor Analysis items that are very difficult to endorse compared to other items can sometimes lead to other ‘factors’ that emerge from the analysis [41]. Because ‘difficulty to endorse’ is taken into account in the Rasch model, the ‘factors’ or ‘dimensions’ that emerge do not reflect difficulty but can be taken to be substantive dimensions of the instrument.

A third way to assess dimensionality is to compare the reliability estimates of two different analyses of the data [39]. The first analysis uses the original items and assumes they are independent. In the second analysis responses to the items that are hypothesised to be dependent are summed into a single polytomous item. If a comparison between the reliability values for the two analyses shows the second analysis to have lower reliability, then the case for multidimensionality is further strengthened. When dependent items were summed into two polytomous items (Polytomous item 1: items 1, 2, 5, 9, 11, 13, 14 and polytomous item 2: items 3, 4, 6, 7, 8, 10, 12, 15, 16) the PSI dropped from 0.56 to nearly 0, and the correlation between the two underlying dimensions was estimated to be 0.

The results of these tests all show that the sixteen items form two dimensions, items that *are not* symptoms of concussion, and items that *are* symptoms of concussion. The logical conclusion is that the items may best be analyzed separately. That was done and the results are described below. However, before a description of those results, results of an investigation of differential item functioning (DIF) are reported. It was expected that the multidimensionality evident in the above results would also manifest in these results.

#### Differential item functioning (DIF) and group means

**Fig. 2:**
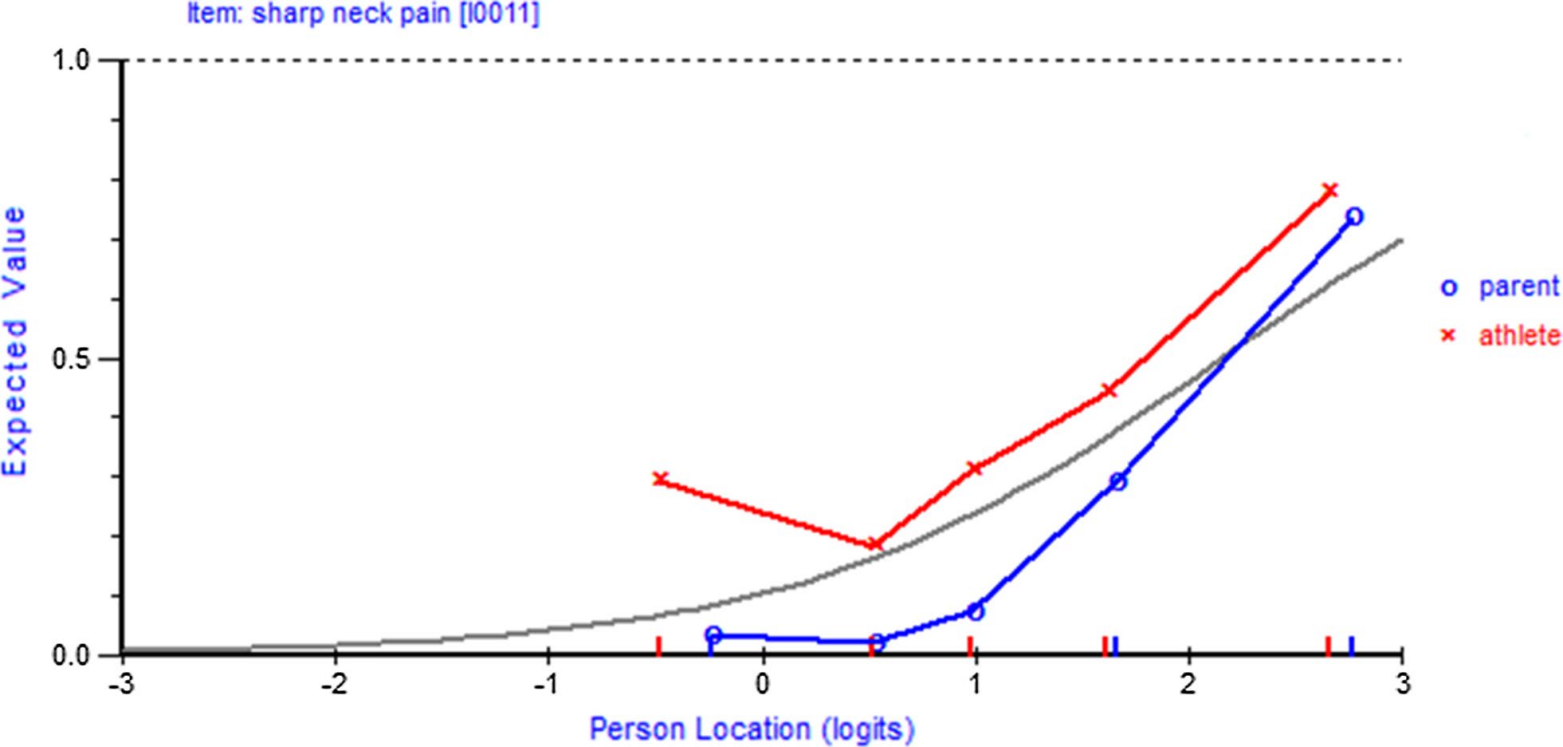
DIF for item 11 *Sharp neck pain*: For the same overall level of awareness of concussion symptoms, athletes identified this item correctly as *not* a symptom of concussion more than parents did

Differential item functioning (DIF) was investigated for the variables Group (*Parents or Athletes*), Gender (*male, female*), Age *(*<*16* *years,* >=*16* *years*), Years Played (H*ow many years you have played organized, coached sports? 1*–*2, 3*–*5, 6*–*10* *years*), and Bellrung (*Have you ever had your ‘bell rung’ when playing organized, coached sports?*). No items showed DIF, except for the variable Group (*Parents or Athletes*). One of these items was item 11 *Sharp pain in neck* and Fig. 2 shows the DIF for item 11 graphically. For the same overall level of awareness of concussion symptoms, athletes identified this item correctly as *not* a symptom of concussion more than parents did.

Interestingly, the items that showed DIF were the items that are *not* symptoms of concussion, that is, items 1 *Abnormal smell*, 2 *Abnormal taste*, 5 *Chest pain*, 9 *Nose bleed*, 11 *Sharp pain in neck*, 13 *Numbness/tingling in arms/hands* and 14 *Weakness in neck range of motion*. Athletes and their parents responded invariantly on the items that *are* symptoms of concussion, that is, items 3, 4, 6, 7, 8, 10, 12, 15, and 16. Andrich and Hagquist [42] distinguished between real and artificial DIF because an item can show DIF that is not real as a result of real DIF in other items. Applying the Andrich and Hagquist [42] procedure confirmed that the DIF in items 1, 2, 5, 9, 11, 13 and 14 can be considered real DIF.

ANOVA showed that athletes’ age, years played and history of concussion had no significant effect on **mean** estimates. An ANOVA revealed that there was no significant difference between the **mean** person estimates of athletes (*M* = 1.157, *SD* = 1.17) and their parents (*M* = 1.072, *SD* = 1.06) (*F*(1,1723) = 1.471, *p* = 0.23). However, studies have shown that comparisons between the summed scores on an instrument for members of subgroups can be misleading if members of the different subgroups respond differently to individual items [40, 45]. Because there is DIF (parents/players) on some of the sixteen symptom recognition items, comparisons between the mean estimates for parents and players may be misleading. One way to ensure valid comparisons is to resolve the DIF by ‘splitting’ the DIF items into two separate items, one for athletes and the other for parents. When the DIF was resolved through splitting the DIF items an ANOVA revealed that there was a significant difference between the mean person estimates of athletes (*M* = −0.191, *SD* = 0.92) and their parents (*M* = 1.341, *SD* = 1.24), *F*(1, 1723) = 388.391, *p* < 0.0001). Parents have a higher mean estimate. Since only the items that *are* symptoms of concussion were responded to by both groups after the other items were split, it reveals that, on these items parents had a higher mean estimate.

#### Separate analyses of symptoms and non-symptoms of concussion

**Fig. 3:**
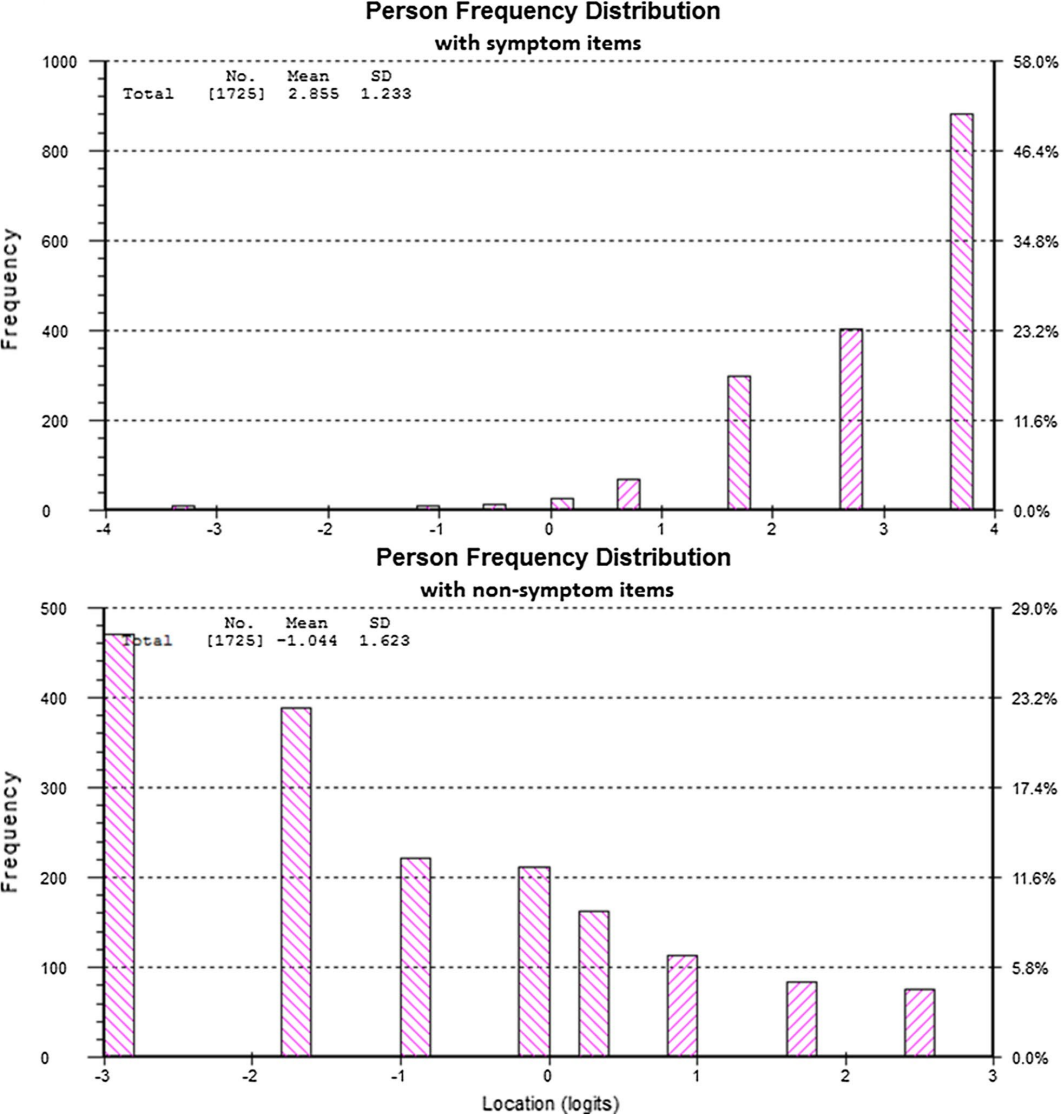
Person distribution from the analysis of items that *are* symptoms of concussion (*top*) and person distribution from the analysis of items that *are not* symptoms of concussion (*bottom*)

Because the results of tests of dimensionality showed that the sixteen items are not unidimensional, items that *are not* symptoms of concussion and items that *are* symptoms of concussion were analyzed separately. Figure 3 shows the person distributions resulting from separate analyses of symptoms and non-symptoms of concussion. On the left is the person distribution from the analysis of items that *are* symptoms of concussion. It is clear from the graph that many persons had a high estimate on these items. On the right is the person distribution from the analysis of items that *are not* symptoms of concussion. On these items many persons had low estimates.

#### Comparison of results of dichotomous and polytomous scoring

The item location estimates resulting from scoring the items dichotomously correlated very highly with those resulting from scoring the items polytomously (r = 0.88). With polytomous scoring Cronbach’s alpha was 0.52, lower than the 0.62 with dichotomous scoring. The PSI, 0.56, was the same as with dichotomous scoring. The same patterns of dimensionality and DIF were found with polytomous scoring. With polytomous items a Rasch analysis provides evidence of whether the response categories functioned as intended, that is, whether scoring *Incorrect*, *Don’t know*, and *Correct* with increasing integers were distinguished and utilized as such by respondents. Once again, there was a difference between symptom items and non-symptom items. For items that are symptoms of concussion the category thresholds were disordered, with the middle category (*Don’t’ know*), not being utilized by respondents. Respondents either knew or did not know whether the item was a symptom, and so the item functioned essentially as a dichotomous item. On the other hand, for the non-symptom items the category thresholds were ordered, with all three categories being used.

## Discussion

The aim of this study was to utilise Rasch measurement theory to examine the psychometric properties of a concussion awareness questionnaire [25] intended for use before and after an education campaign regarding concussion associated with AF. This is vital as players and parents need to aware of possible signs and symptoms of SRC in circumstances where medical care at games and training may be limited. Because the questionnaire is to be used to assess change in players’ and their parents’ awareness of SRC symptoms after a targeted education campaign it needs to be valid, reliable and able to detect clinically and statistically significant change.

The use of self-report concussion symptom scales has been criticized as unreliable because athletes subjectively report their symptoms and may be motivated to not report symptoms in order to hasten return to play (RTP) [34]. However, an even bigger criticism is highlighted by Alla et al. [13] who noted that most scales have not been psychometrically validated. Having sound psychometric properties for the symptom scales is important because these tools are often used to track symptom resolution and aid the clinician in making the important and complex RTP decision [14].

The literature evaluating the psychometric properties of self-report scales is limited. In Alla et al. [13] systematic review, only 7 of the 20 scales had any published psychometric properties, and none of the symptom scales were supported by a complete set of published psychometric properties, including item selection, reliability, validity, sensitivity, specificity, and change scores. Of the studies evaluated by Alla et al. [13], only the Post-Concussion Scale: ImPACT-22 item (construct), SCAT Post-Concussion Symptom Scale (face, content), Head Injury Scale 16-item (factorial), and Head Injury Scale 9-item (factorial, construct) have been evaluated for validity. Recently, the Standardized Assessment of Concussion (SAC) [46] as assessed using classic item analysis and Murray et al. assessed the reliability and validity evidence for detecting balance disturbance in athletes with a concussion [47]. Neither used Rasch measurement theory. The scales listed are for clinical use only and not utilized in a non-clinical setting such as a parent and player concussion educational opportunity and furthermore, the Rasch measurement model was not utilised. Therefore, the innovative use of the Rasch measurement theory in the psychometric analysis of a symptoms scale for use in a non-clinical setting such as a community educational opportunity is unique.

There are at least three ways the items from the instrument could be scored for analysis: (i) dichotomous scoring where *Don’t know* is scored as *Incorrect*, (ii) polytomous scoring where respondents get partial credit for *Don’t know*, that is, for being aware they don’t know whether something is a symptom, and (iii) scoring *Don’t know* as missing data. Preliminary analyses indicated that with option iii the reliability was too low because of the missing responses. The results from scoring the responses polytomously (ii) were similar to the results with dichotomous scoring (i), but there were also some differences.

The main finding from this research, which concentrated on the sixteen items of the Symptom Recognition section of the questionnaire, is that these items measure two ‘dimensions’ or ‘factors’ in concussion symptom recognition. The first ‘dimension’ consists of those items that *are* symptoms of concussion, items 3 *Loss of memory*, 4 *Blurred vision*, 6 *Dizziness*, 7 *Confusion*, 8 *Headache*, 10 *Loss of consciousness,* 12 *Nausea,* 15 *Sleep disturbance* and 16 *Problems studying/class work.* The second dimension consists of items that *are not* symptoms of concussion, items 1 *Abnormal smell*, 2 *Abnormal taste*, 5 *Chest pain*, 9 *Nose bleed*, 11 *Sharp pain in neck*, 13 *Numbness/tingling in arms/hands* and 14 *Weakness in neck range of motion*. Respondents’ estimates on these two dimensions had a zero correlation. This could be due to several reasons. For example, items 3, 4, 6, 7, 8, and 10 relate to the head region, thus associated with head-trauma events such as concussion. Furthermore, players and parents with previous experience playing sport may have heard these items mentioned by coaches and medical staff as those who have had personal experience with contact sports score ‘higher’ on almost every measure with respect to concussion knowledge than those who have never had personal contact sport experience [48]. Finally, recent investigation [49] found that most students recognised headache, dizziness and confusion as signs of a concussion and many learned about concussion from their parents, at school or online, others who have read or saw information about the topic in the media (i.e., articles, blogs, or documentaries) [48].

The symptoms of concussion were easy to correctly identify, whereas it was more difficult to correctly identify the items that *are not* symptoms of concussion. In Factor Analysis items that are very difficult to endorse compared to other items can sometimes lead to difficulty ‘factors’ that emerge from the analysis [41]. Because ‘difficulty to endorse’ is taken into account in the Rasch model, the two factors or dimensions found in this study do not reflect difficulty factors but can be taken to be substantive.

What do the above findings mean for the measurement of change? Parents and players were able to identify most or all of the symptoms of concussion, so one would not expect pick up any positive change on these items after an education campaign. Parents and players were not as good at distinguishing symptoms that *are not* symptoms of concussion. It is on these items that one may possibly expect improvement to manifest, so to evaluate the effectiveness of an education campaign it would pay to look for improvement in distinguishing symptoms that *are not* symptoms of concussion. If the sum score of all sixteen items were used to measure change any possible improvement could be masked or ‘diluted’ by responses to items that *are* symptoms of concussion, whereas if a sum score only on the items that *are not* symptoms of concussion is analyzed pre and post intervention, especially if scored polytomously, change would be more readily detected.

In a separate paper by the same authors [50], in which the same set of responses were analyzed using traditional methods, no difference was found between parents and athletes’ recognition of concussion symptoms. Analyzing these responses using the Rasch model revealed differential item functioning on certain items for parents and athletes. The items that showed DIF were the items that are *not* symptoms of concussion. When the DIF was resolved a significant difference was found between parents and athletes. Parents were better than athletes at identifying the symptoms of concussion. Person fit to the model confirmed that these findings were not due to response biases.

Athletes’ age, years played and history of concussion had no significant effect on concussion symptom recognition. These findings are similar to findings described in a separate paper by the same authors [50], in which responses were analyzed using traditional methods.

There were several limitations in this study. Firstly, there was a low response rate. This was anticipated due to the use of a web site link, as it was difficult to schedule face-to-face captive audience gatherings over the state of Western Australia. Secondly, there was a disproportionally greater number of parents responding than players. Equal numbers in the two groups would have been more ideal, especially for the DIF analysis. Lastly, although this study investigated the general performance of the ‘Symptom recognition’ section of the questionnaire, it did not make comparisons with scores from other measures of concussion symptom recognition or alternative measures of concussion awareness, which is an important aspect of convergent construct validity.

## Conclusions

The primary aim of concussion tools available such as the one analysed in this study usually center on pre- and post-measurement of an educational opportunity used to increase knowledge on SRC. The innovative use of the Rasch measurement theory in the psychometric analysis in this study indicated that when using this concussion tool an educational campaign should focus primarily on distinguishing symptoms that *are not* symptoms of concussion in order to benefit both parents and players. Although there is minimal evidence supporting concussion educational opportunities, awareness and knowledge of concussion is the greatest positive influence for symptom reporting among young athletes [51]. Often players do not know how a concussion could affect them and sometimes do not think it warrants reporting, but concussion education helps young athletes identify what qualifies as a concussion [51]. However, when selecting a concussion tool, it is important to use one that has undergone psychometric evaluation to properly develop and measure an educational awareness opportunity.

## Abbreviations

SRC: sports-related concussion
AF: Australian football
S&S: signs and symptoms
RUMM2030: Rasch Unidimensional Measurement Model software
PCA: principal component analysis
DIF: differential item functioning
